# PD−L1 immunostaining: what pathologists need to know

**DOI:** 10.1186/s13000-021-01151-x

**Published:** 2021-10-25

**Authors:** Mohammed Akhtar, Sameera Rashid, Issam A. Al-Bozom

**Affiliations:** Department of Laboratory Medicine and Pathology, Hamad Medical Corporation, P.O. Box 3050, Doha, Qatar

**Keywords:** Cancer, Immune cells, PD-L1, PD-1, Immune checkpoint, T-cells, Inhibitors, Immunohistochemistry, Activation, Inhibition

## Abstract

**Background:**

Immune checkpoint proteins, especially PD-L1 and PD-1, play a crucial role in controlling the intensity and duration of the immune response, thus preventing the development of autoimmunity. These proteins play a vital role in enabling cancer cells to escape immunity, proliferate and progress.

**Methods:**

This brief review highlights essential points related to testing for immune checkpoint therapy that histopathologists need to know.

**Results:**

In recent years, several inhibitors of these proteins have been used to reactivate the immune system to fight cancer. Selection of patients for such therapy requires demonstration of PD-L1 activation on the tumor cells, best done by immunohistochemical staining of the tumor and immune cells using various antibodies with predetermined thresholds.

**Conclusions:**

Immune checkpoint therapy appears to be promising and is rapidly expanding to include a large variety of cancers.

## Introduction

**Fig. 1:**
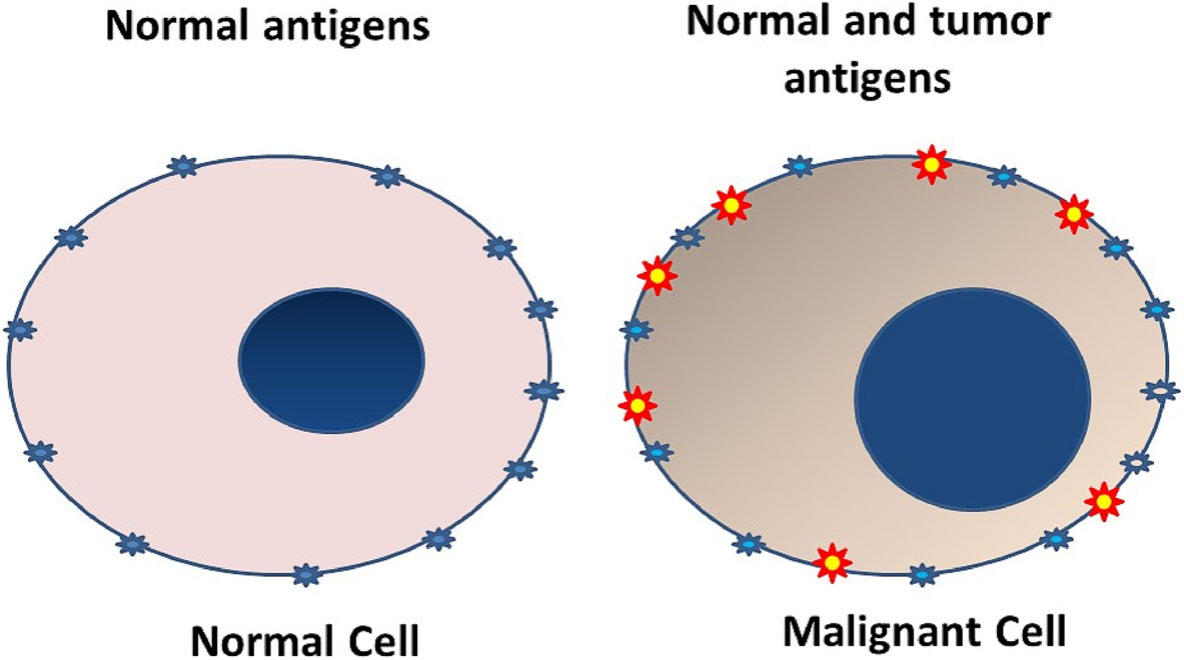
Cartoon showing surface antigens on a normal cell, compared with a cancer cell which has the normal antigens as well as additional cancer-specific and cancer-related antigen (shown as yellow-red)

**Fig. 2:**
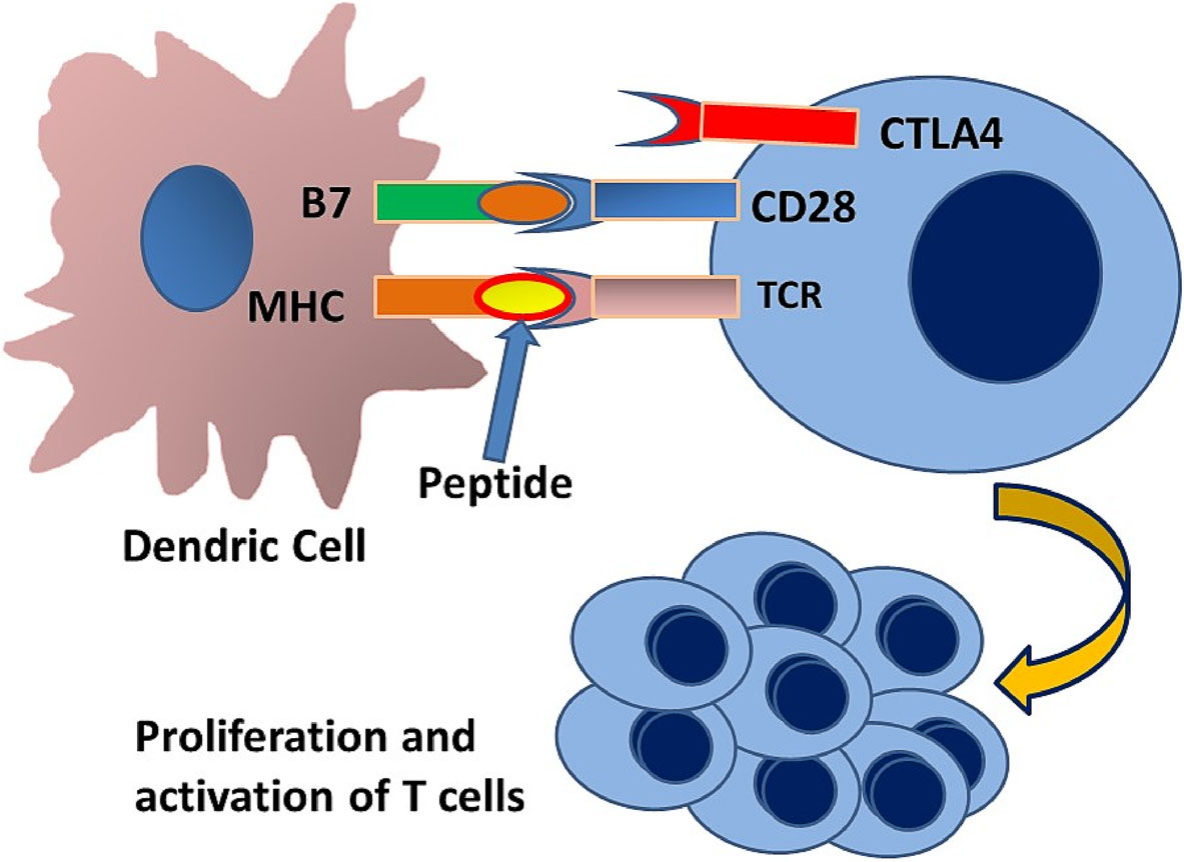
Dendritic cells capture and process the cancer antigens and present these as peptides in association with MHC on the surface of the cells. The T-cell receptor on T- lymphocytes interacts with MHC and the peptide. In addition to binding to antigen-loaded MHC, T cells require a secondary signal to become activated. CD28 on the lymphocyte binds to the B7 on the surface of the dendritic cell. This interaction causes the T-cell to undergo stimulation and multiplication to become activated T-cells (ATC)

Tumor cells have surface antigens like those present on normal cells. However, they also manifest additional antigens that are either absent or expressed in minimal quantities on non-neoplastic cells. These are called tumor-specific or tumor-associated antigens (Fig. 1). Tumor antigens are present as peptides of approximately 8-10 amino acids along with major histocompatibility complex (MHC) class I and 2. Dendritic cells within tumor or in the regional lymph nodes capture these peptides and present them to the cytotoxic CD8+ T-cells resulting in proliferation and activation of these cells (Fig. 2). Activated cytotoxic T cells (ATC) return from general circulation and infiltrate the tumor microenvironment. These target and destroy tumor cells with the corresponding tumor antigen [1, 2].

### Immune checkpoint regulators

**Fig. 3:**
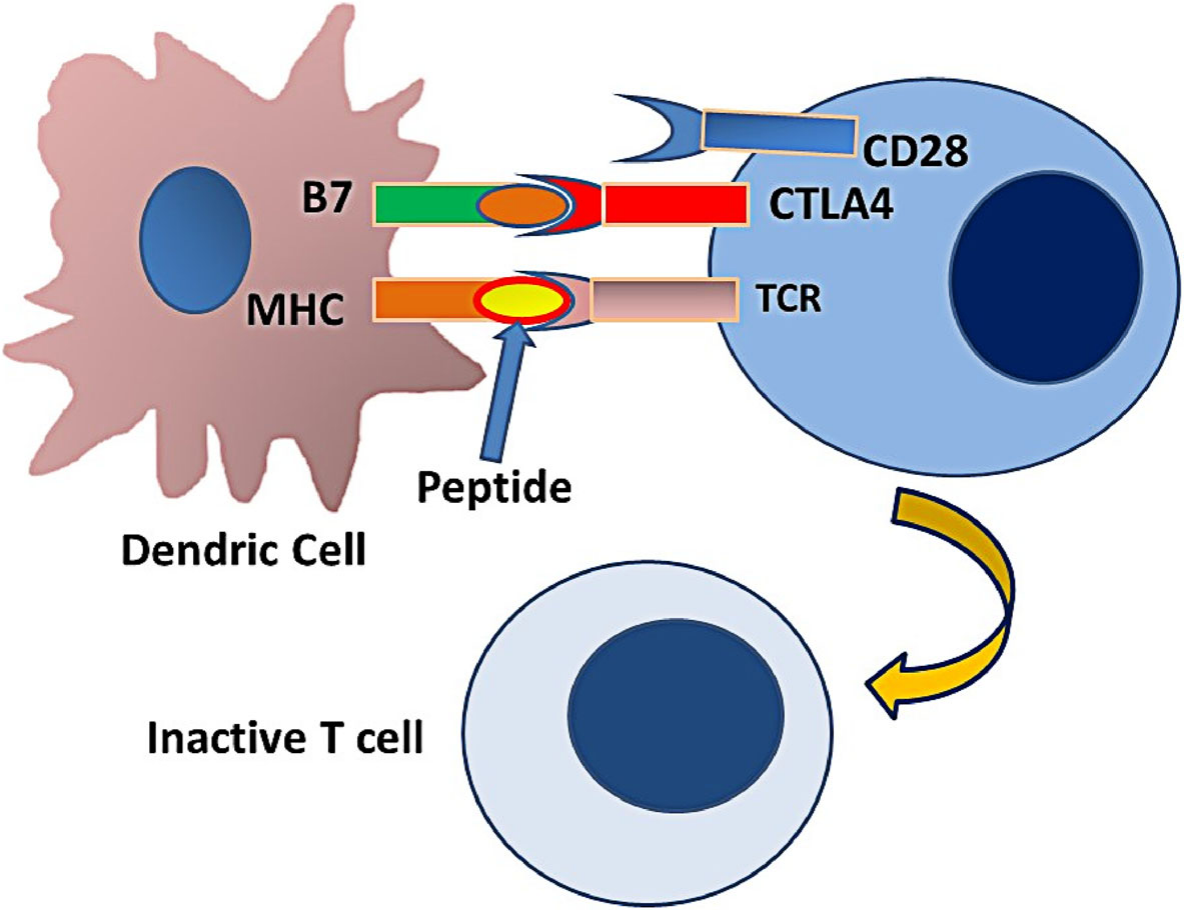
CTLA4 on the surface of ATC competes with CD28 for interaction with B7. This interaction causes the T-cell to undergo regression and inactivation

Under normal conditions, the immune system functions to protect the host against infectious diseases and tumors. In addition, it plays a vital role in clearing the body of unhealthy and ailing cells. An overactive immune system, however, may cause autoimmunity resulting in variable tissue damage. Immune checkpoints are inhibitory regulators of immune system that are crucial for maintaining self-tolerance and preventing autoimmunity by controlling the duration, extent, and intensity of immune response to minimize collateral tissue damage. One such checkpoint is cytotoxic T-lymphocyte-associated protein 4(CTLA4). It is constitutively expressed in regulatory T cells (Tregs) and is upregulated in activated conventional T cells. CTLA4 bears similarity to T-cell costimulatory protein CD28, and both molecules compete to bind to CD80 (B7). CTLA4, when activated by CD80, transmits an inhibitory signal to T cells, whereas CD28 transmits a stimulatory signal (Figs. 2 and 3). Another pair of cell surface proteins, namely programmed cell death 1 (PD-1)/programmed cell death ligand 1 (PD-L1), plays an essential role in normal immune checkpoint function [3, 4].

### PD-1 and PDL-1

**Fig. 4:**
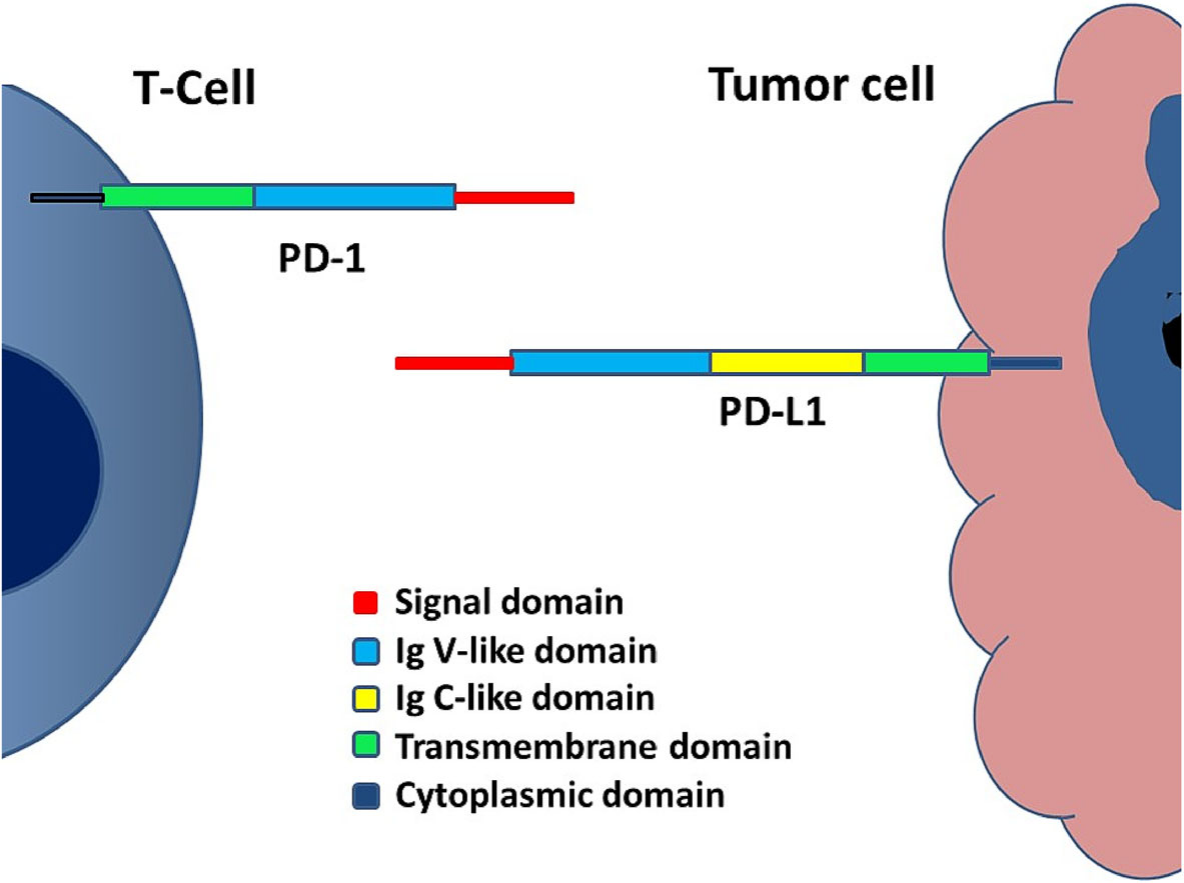
Cartoon revealing the structure of PD-L1 on the tumor cell and PD-1 expressed on the surface of ATC

PD-1 (CD279) is a 288-amino acid type I transmembrane protein predominantly expressed on antigen-experienced memory T cells in peripheral tissues and less commonly on B cells, activated monocytes, dendritic cells (DC), and natural killer (NK) cells. PD-1 is homologous to the B7 family of protein receptors and is composed of immunoglobulin V (IgV)-like extracellular domain and an intracellular domain that contains two phosphorylation sites (Fig. 4).

**Fig. 5:**
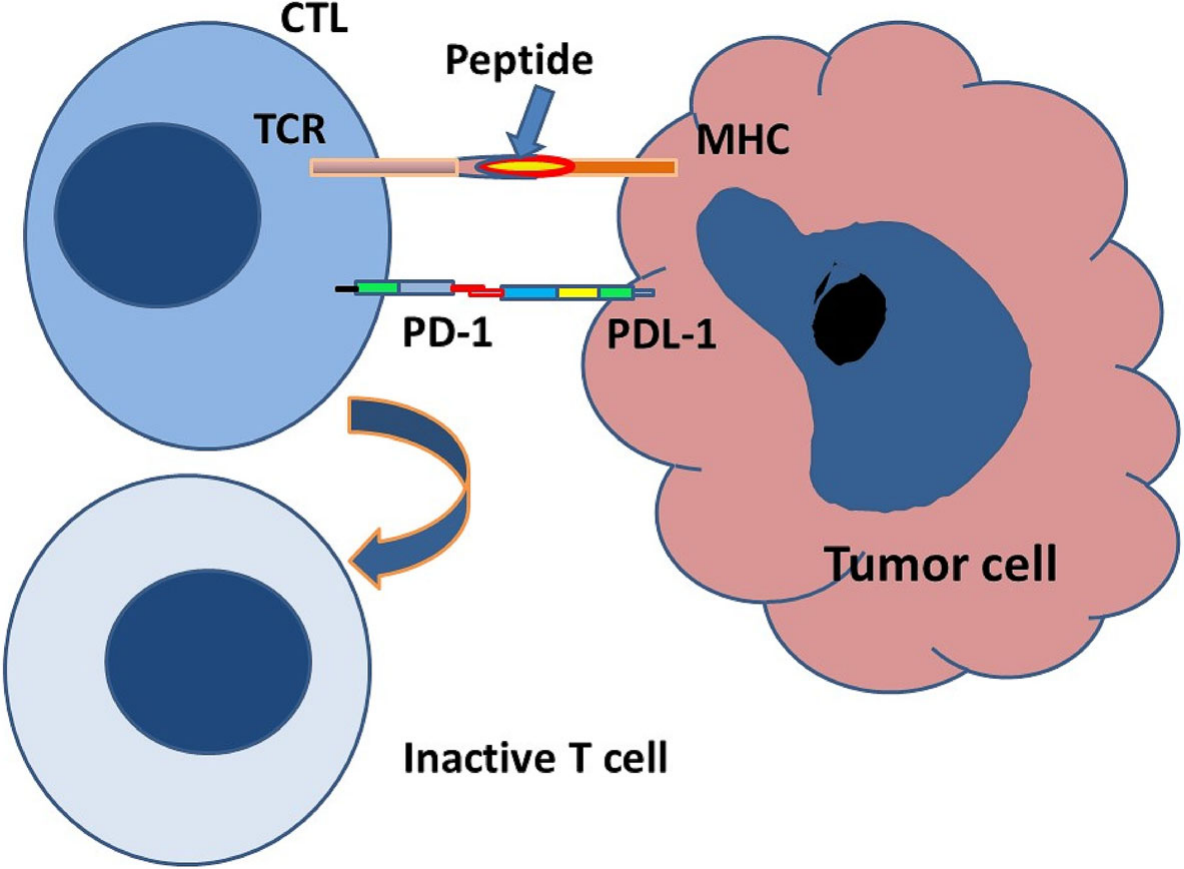
Engagement of PD-L1 on the tumor cell with PD-1 on the ATC along with co-stimulation provided by T-cell receptor and MHC results in inactivation of the lymphocyte

PD-L1 (B7-H1 or CD274) is a 290- amino acid protein, member of the B7 family of type I transmembrane protein receptors, including two extracellular domains, IgV-like and IgC-like domains; a transmembrane domain; and a cytoplasmic domain (Fig. 4). This protein is expressed on many types of cells including antigen-presenting cells (APCs), T cells, B cells, monocytes, and epithelial cells. After activation in response to pro-inflammatory cytokines, these cells upregulate the expression of PD-1 [3–5]. In addition, the binding of PD-L1 to PD-1 activates the downstream signaling of PD-1 receptor in T cells, thus inhibiting the proliferation, cytokine generation, release, and cytotoxicity of T cells (Fig. 5).

The physiological role of immune checkpoints is to prevent harmful immune attack on self-antigens during an immune response. Each checkpoint pathway decreases immune activation through intracellular signaling mechanisms and thus negatively regulates the effector immune cells by inducing T cell regression and exhaustion. Inhibitory checkpoint proteins, including programmed PD-1, PD-L1, or CTLA-4, can suppress antitumor T-cell responses [5, 6]. Enhancement of these checkpoint proteins is a common strategy of several solid tumors such as non-small cell lung carcinoma, malignant melanoma, or urothelial carcinoma. By upregulating PDL1 expression, these tumors use many of these pathways as crucial mechanisms to deactivate the CTLs and thereby escape antitumor immune responses.

**Fig. 6:**
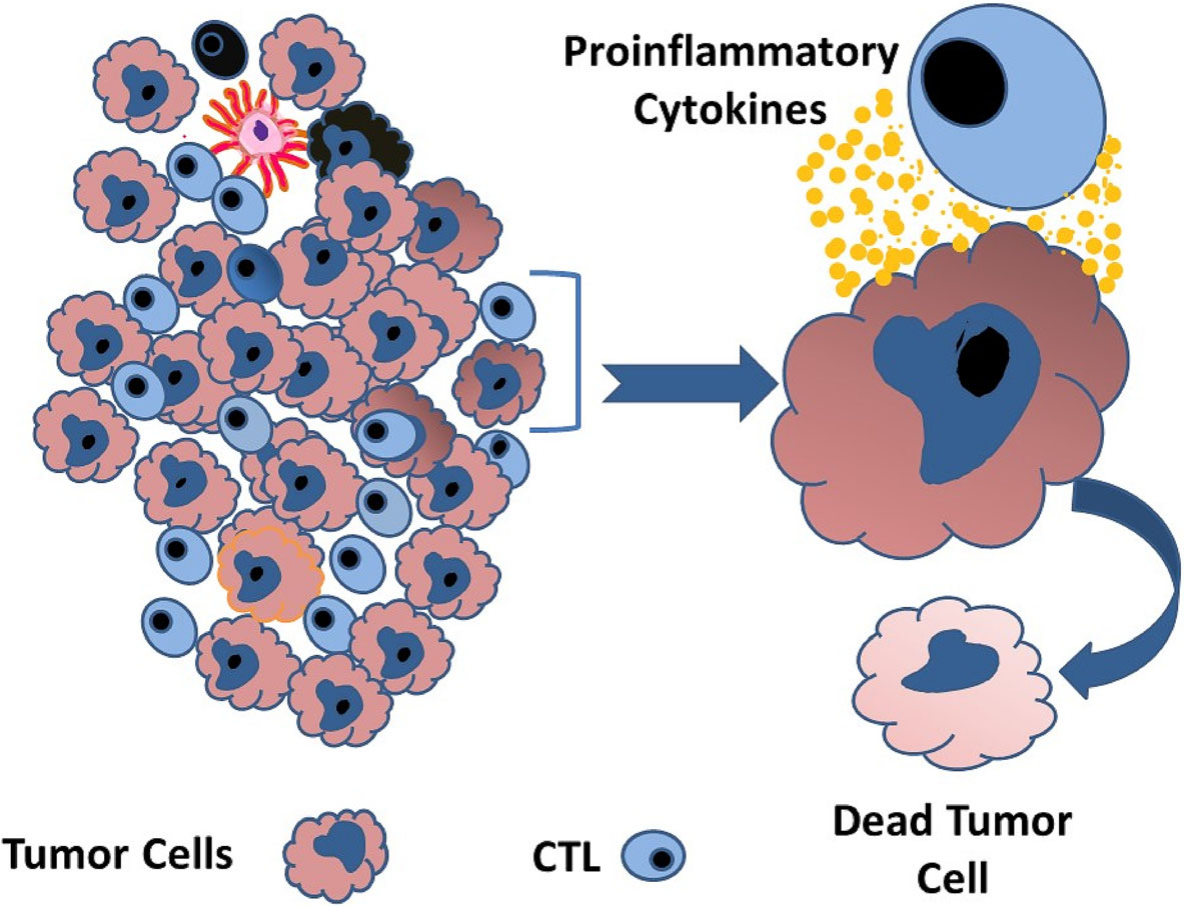
Activated lymphocytes invade the tumor microenvironment and seek cancer cells for destruction. To kill the cancer cells, ATC produces pro-inflammatory cytokines, which may accumulate and alter the tumor’s micro-environment. As a result, cancer cells increase the expression of PD-L1 on the cell surface

**Fig. 7:**
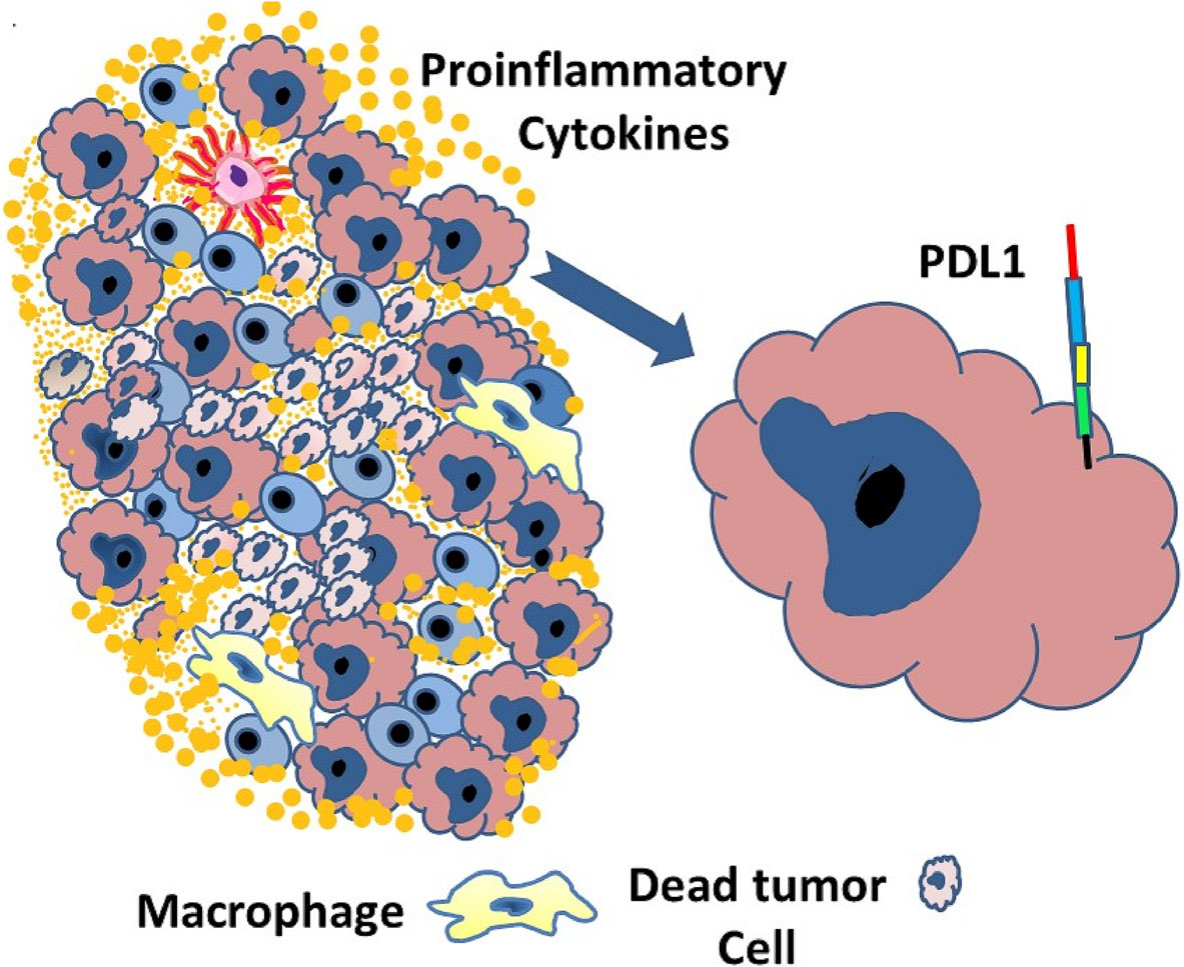
Interaction of PD-L1 on cancer cells with PD-1 on the lymphocytes results in inactivation of the lymphocytes and increased numbers of Tregs ,thus providing an immune inhibitory environment

**Fig. 8:**
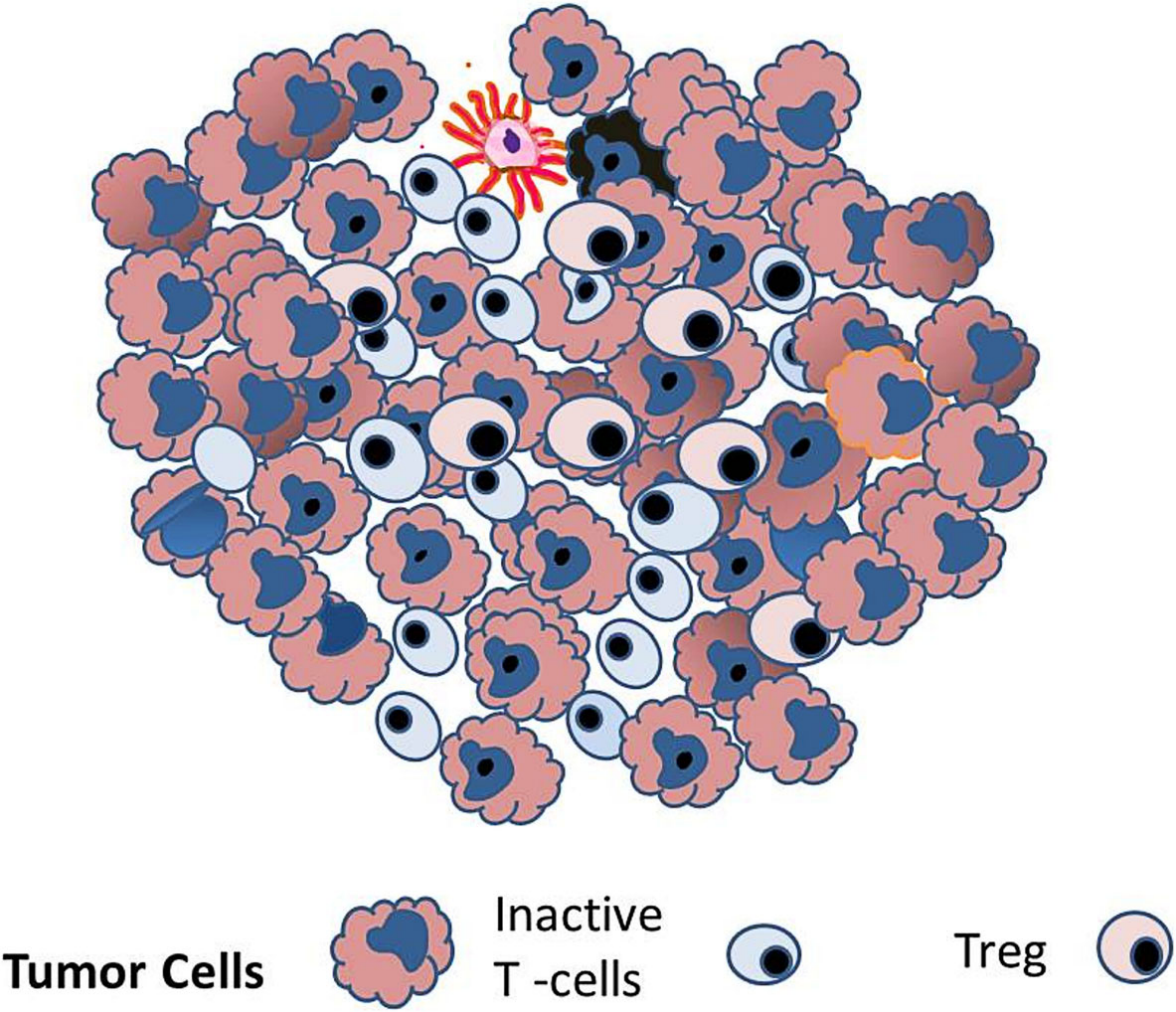
As a consequence of the immune inhibitory environment, cancer cells can bypass the immune system, proliferate and undergo progression. Inactivated T-cells persist and are kept in an inactive state by Tregs

Activated T cells infiltrate the tumor to destroy tumor cells (Fig. 6). During interaction between tumor cells and ATC, several pro-inflammatory cytokines (IFNγ, TNF-α, IL-4, and IL-2) are released in the tumor microenvironment resulting in immunomodulation of the tumor cells, with upregulation of PD-L1expression on their surface (Fig. 7). In addition, tumor-infiltrating immune cells, including ATCs, antigen-presenting cells, and dendritic cells also upregulate the expression of PDL1 on their surface. PD-1 is constitutively present on the surface of ATCs. As PD-L1 present on the tumor cells and several immune cells engages with PD-1, it transmits a negative costimulatory signal causing ATCs to undergo inactivation, dormancy, and regression. These changes enable the tumor to bypass the immune system and ultimately progress, disseminate, and metastasize. These deactivated T-cells remain inhibited but persist in the tumor microenvironment. The pro-inflammatory microenvironment also attracts Tregs that help maintain the T-cell regression and dormancy (Fig. 8). These changes enable the tumor cells to bypass the immune system and undergo progression [5–9].

A tumor can become positive or negative for surface PD-L1 through several biological processes. Tumor-infiltrating T cells induce the tumor cells to express PDL-1; the absence of T cells may lead to a lack of reactive PD-L1 expression. Genetic mechanisms may also determine constitutive PD-L1 expression. There may be genetic events within the tumor cells that preclude PD-L1 expression upon T cell infiltration. Thus, the presence or absence of cancer cell surface PD-L1 may have different functional meanings and treatment implications depending on the underlying mechanism of expression [10].

### Immune check point inhibitors

Immunotherapy drugs called immune checkpoint inhibitors block the binding of checkpoint proteins with their partner proteins, thus preventing the inhibitory signal from being sent to the T-cells, allowing them to remain activated to kill cancer cells. The U.S. Food and Drug Administration (FDA) approved the first immune checkpoint inhibitor, ipilimumab, in 2011 to treat melanoma as an inhibitor of CTLA 4 (Bristol-Myers Squibb). In 2014 the first anti-PD-1 antibody, pembrolizumab (Merck), was approved by the FDA for use in metastatic melanoma. Since then, FDA has approved additional therapeutic monoclonal antibodies that target either PD-1 or PD-L1. These agents are available to treat various tumors, including Merkel cell carcinoma, Hodgkin lymphoma, non-small cell lung, renal, urinary bladder, head and neck, gastric and hepatocellular carcinoma, among others [5–9]. Some of these checkpoint inhibitors block PD1 ( Pembrolizumab, Nivolumab, Cemiplimab), while others inhibit PDL1 ( Atezolizumab, Avelumab, Durvalumab).

The development of immune checkpoint inhibitors has changed treatment paradigm for advanced cancers across many tumor types. Despite encouraging and sometimes remarkably durable responses in a subset of cases, most patients fail to respond. Interestingly, occasional patients in the PD-L1-negative subgroup may also benefit substantially from such therapy. Tumors have adopted the PD-1/PD-L1 axis for an immune escape to facilitate growth and progression but it may also serve as a potential therapeutic target for immune checkpoint inhibitors. These therapeutic agents, however, are very costly and may have significant side effects. Therefore, it is imperative to evaluate the tumor for increased expression of PDL1 before initiation of therapy. On this basis, PD-L1 protein expression on tumor or immune cells has emerged as the potential predictive biomarker for sensitivity to immune checkpoint blockade therapy [7–11].

### Determination of PDL1 expression

PDL1 expression may potentially be recognized and measured by a variety of available diagnostic techniques. For example, in advanced cancer, plasma PD-L1 protein levels could provide means for monitoring of PDL1 expression. Enzyme-linked immunosorbent assay (PDL1-ELISA) can potentially analyze PDL1 quantitatively or qualitatively in plasma. In addition, western blot might help to detect specific proteins in tissue homogenate. Mutational findings from targeted NGS panels and study of messenger RNA and micro RNA may also provide valuable insight into the level of expression of various components of the crucial immune checkpoints in patients with cancer [12, 13]. However, immunohistochemistry is the only widely available, practical and economical approach for studying PD-L1 expression in a tumor. Furthermore, this technique helps identify patients who may be more likely to benefit from immunotherapy with PD-1/PD-L1 inhibitors.

Many commercially available PD-L1 immunohistochemical tests are available to select patients for treatment with checkpoint inhibitors. The clinical trials that led to FDA approval of these agents used different immunohistochemical platforms with various PD-L1 antibodies to assess PDL1 expression on tumor cells, tumor-infiltrating immune cells, or both. Four PD-L1 immunohistochemical assays registered with the FDA used four different PD-L1 antibodies (22C3, 28–8, SP263, SP142) on two IHC platforms (Dako and Ventana), each with a specified scoring system. These PD-L1 antibody clones are available as prepackaged kits for use on the approved platform. The clinical trials leading to their approval used specific immune checkpoint inhibitors with specific diagnostic assays. Each of these assays uses a unique antibody with proprietary reagents, protocols, and thresholds for defining the positive expression of PD-L1 [14–16].

These trials used two types of assays: First, companion diagnostic assays, which provide required and essential information for safe and effective use of the corresponding drugs. The second group comprises complementary assays, which may be helpful but do not have a critical role in selecting patients for specific drug therapy. For example, PD-L1 IHC22C3 pharmDx tests mostly have status as companion diagnostics. On the other hand, PD-L1 IHC 28-8 pharmDx, PD-L1, Ventana PD-L1 SP142, and Ventana PD-L1 SP2632 testing are complementary diagnostics [17–19]. However, the most recently approved companion assay uses PDL1 IHC28-8 pharmDx for metastatic non-small cell lung cancer (Table 1).

**Table 1:** Details of FDA-approved immune checkpoint inhibitors and corresponding antibodies for immunohistochemical staining

	Year	Drug	Target protein	Antibody	Threshold %	Cell type	Therapy type
NSCLC	2015	Pembrolizumab	PD-1	22C3	50	TC	2nd line
NSCLC	2016	Pembrolizumab	PD-1	22C3	1	TC	2nd line
NSCLC	2016	Pembrolizumab	PD-1	22C3	5	TC	1st line
Bladder CA	2016	Atezolizumab	PD-L1	SP142	10	IC	1st line
Bladder CA	2017	Pembrolizumab	PD-1	22C3	5	IC	1st line
Bladder CA	2017	Durvalumab	PD-L1	SP2632	10	IC+TC	1st line
Gastric CA	2018	Pembrolizumab	PD-1	22C3	1	IC+TC	3rdline
Cervical CA	2018	Pembrolizumab	PD-1	22C3	1	IC+TC	2nd line
Triple negative Breast CA	2019	Atezolizumab	PD-L1	SP142	1	IC	1st line
NSCLC (Metastatic)	2020	Atezolizumab.	PD-L1	SP142	TC:50 IC: 10	IC+TC	1st line
NSCLC (metastatic)	2020	Nivolumab + ipilimumab	PD-L1	28-8	1	TC	1st line

Additional less expensive antibodies are available for PD-L1 staining that employ different staining platforms and protocols with varying systems of scoring and thresholds for predictive evaluation. Standardizing and validating these biomarker tests is required by using exclusively prepackaged test kits of reagents running on company-specific staining platforms. Recent studies indicate that several assays (Dako 22C3, Dako 28-8, Ventana SP263) can be used interchangeably in multiple settings except for the Ventana SP142 assay, which detected significantly fewer tumor cells than other assays [17]. Free antibody clones such as Abcam 28-8, Cell Signalling E1L3N, and others are less expensive than prepackaged counterparts. These have been suggested as possible alternatives and validated on different staining platforms without quality impairment [18]. PD-L1 tests must be reproducible, both the technical procedure of staining and the interpretation of the test by pathologists. Pre-analytical issues such as tissue fixation and processing significantly impact the outcomes of immunohistochemical reactions and might affect the result of different PD-L1 IHC tests [17–20].

### Interpretation and reporting

**Fig. 9:**
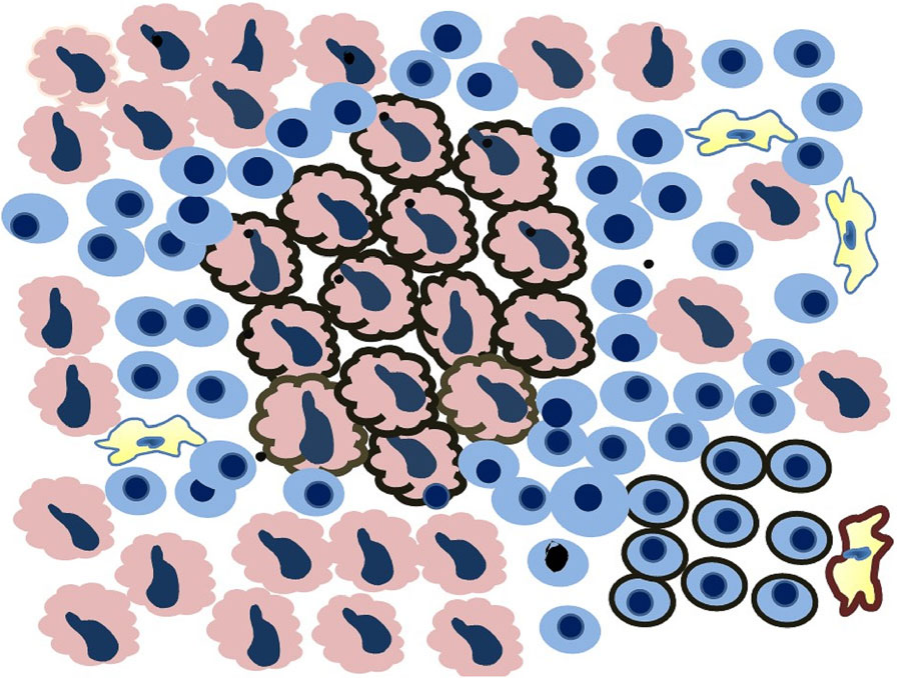
Schematic drawing of a tumor with PD-L1 staining. There are 37 tumor cells, 14 of which are depicting membrane staining (middle part of the drawing). In addition, 10 of the tumor immune cells, including one macrophage, are positive for PD-L1 (lower right-hand corner). Based on this, tumor positive score (TPS) and combined positive score (CPS) can be calculated. $\text{TPS}=\frac{(No.positvetumorcells)14}{\left(No.viabletumorcells\right)37}\times100=37.8$ $\text{CPS}=\frac{(No.allpositvecells)24}{\left(No.viabletumorcells\right)37}\times100=64.8$

**Fig. 10:**
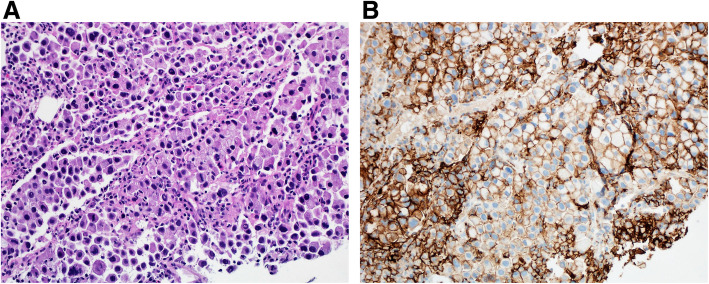
**A** Case of pulmonary adenocarcinoma, solid type. **B** Immunohistochemical staining for PD-L1 showing heterogeneous staining of tumor cells ranging from 1+ to 3+ intensity. TPS: 100. (DAKO 22C3 antibody)

**Fig. 11:**
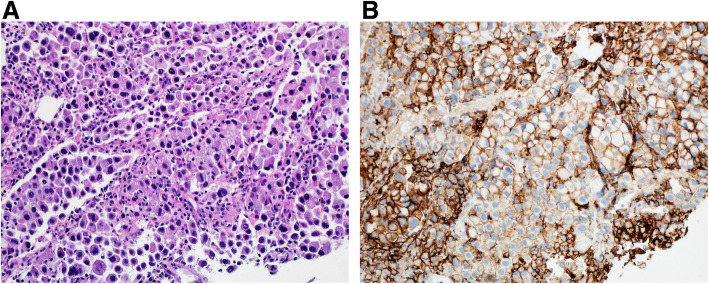
**A** Case of metastatic pulmonary adenocarcinoma to the liver. **B** Immunohistochemical staining for tumor cells, moderate to intense staining (2+-3+) for PD-L1 TPS: 90 (DAKO 22C3 antibody)

**Fig. 12:**
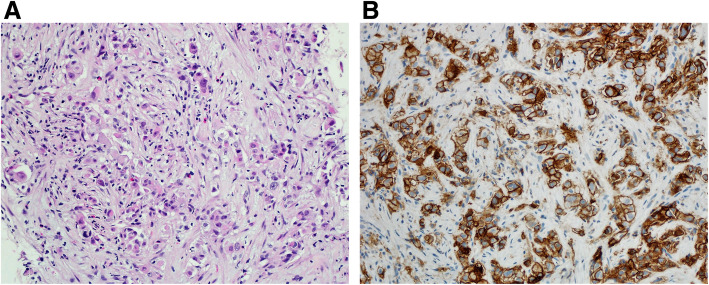
**A** Case of buccal mucosa squamous cell carcinoma. **B** Immunohistochemical staining for PD-L1 revealing staining of tumor cells (right side of the figure) and tumor immune cells (left side of the figure). CPS: 90 (DAKO 22C3 antibody)

**Fig. 13:**
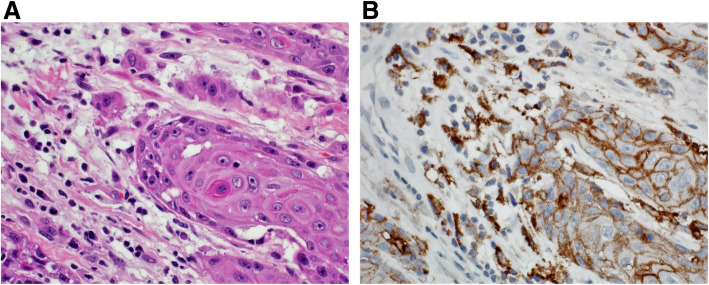
**A** Case of poorly differentiated gastric adenocarcinoma. **B** Immunohistochemical staining of the tumor showing staining of tumor immune cells while tumor cells are predominantly negative. CPS: 20. (DAKO 22C3 antibody)

**Fig. 14:**
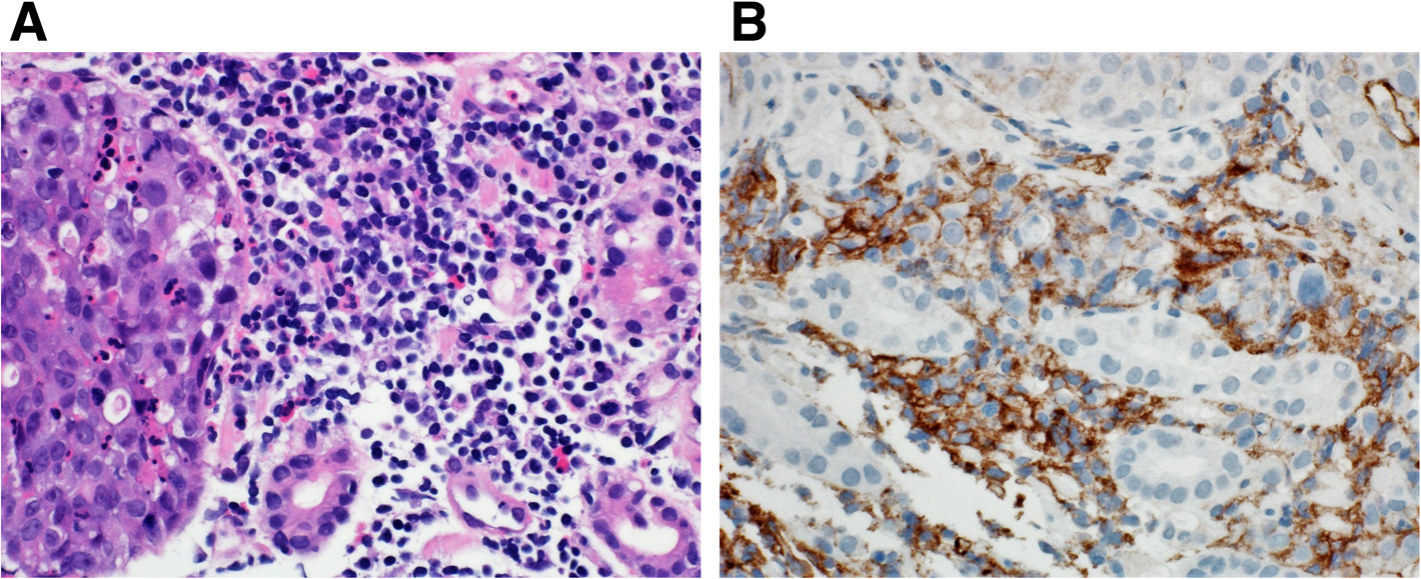
**A** Case of moderately differentiated gastric adenocarcinoma. **B** immunohistochemical staining of the tumor in which only tumor cells are positive for PD-L1; while tumor immune cells are negative. CPS: 80. (DAKO 22C3 antibody)

**Fig. 15:**
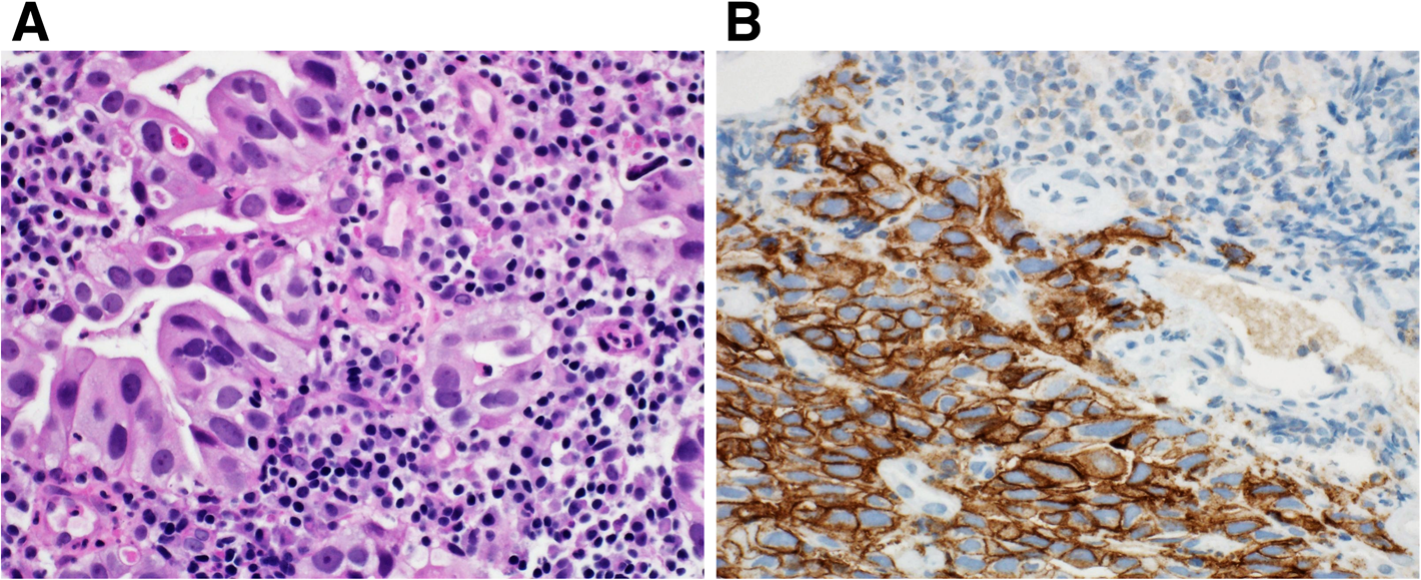
A diagram depicting expression of PD-L1 positive tumor infiltrating immune cells (marked by red border) in an area of tumor cells (marked by black dotted line). The proportion of tumor area occupied by PD-L1-positive immune cells of any intensity determines the immune cell (IC) score

Tumor cells that manifest membranous staining of any intensity are considered positive. Developing clinically relevant and reproducible scoring method for PD-L1 for identifying patients who will respond effectively to anti-PD-1 therapy is the key for establishment of companion or complementary diagnostic assays. The scoring method for PD-L1 IHC 22C3 PharmDx in NSCLC consists of capturing the percentage of stained tumor cells designated as tumor proportion score (TPS), which works well with non-small cell lung cancer (Figs. 9, 10 and 11). However, in subsequent protocols for treating gastric and other cancers, TPS was not efficient in identifying responders because it did not include tumor-infiltrating immune cells in calculating the score. Moreover, additional data indicates that PD-L1 staining on both tumor and tumor-associated immune cells has superior correlation with clinical outcome in some tumors. Therefore, a method for evaluating both cancer and immune cells in one area using the combined positive score (CPS) was developed [9, 14, 15]. CPS depends on the number of PD-L1 positive cells (including tumor cells, lymphocytes, and macrophage) in relation to total viable tumor cells, allowing quantification of tumor and immune cells in a single reading (Figs. 12, 13 and 14). A third method uses percentage of PD-L1 expression in tumor-infiltrating immune cells (IC) assessed as the proportion of tumor area occupied by PD-L1 positive immune cells of any intensity in any tissue compartment (Fig. 15). In tumor-associated immune cells, membrane or cytoplasmic staining is considered positive [19, 20].

### Use of cytological material

None of the original clinical trials for validation of PD-L1 testing used cytology specimens. However, several subsequent studies using paired histology and cytology specimens were published. These studies showed that adequately cellular cytological cell blocks (more than 100 tumor cells) may be suitable for PD-L1 testing and can provide equally reliable results [21, 22]. This avenue may be advantageous when histologic material is not feasible due to the patient’s condition or other circumstances. In such cases, the pathologist may establish the cancer diagnosis and provide PDL-1 test results based on cytology alone. Most published studies used cytologic material from non-small cell lung cancer obtained by endobronchial ultrasound-guided needle aspiration (EBUS-FNA) or effusion fluids. Caution is required, however, when considering the use of practices not formally validated in clinical trials to ensure that patients receive appropriate and effective treatment.

### Artifacts and pitfalls

Tumor cells with complete or incomplete membrane staining are considered positive; however, in a gland-forming tumor, staining limited to the luminal border is negative. In tumor-infiltrating cells, membrane, as well as cytoplasmic staining, is considered positive. Histiocytes/macrophages in various body sites may express PDL-1. Macrophages within glandular lumens may be strongly positive; however, it is regarded as a negative result if there is no staining in tumor cells. Bacteria and acellular debris may have significant positivity and should not affect stain interpretation. Intracellular pigments such as melanin, hemosiderin, and anthracosis can complicate the interpretation of staining.

Furthermore, as platelets express PD-L1, their aggregation in debris or tissue may impart false positivity. Nuclear staining may rarely be encountered but is not part of the scoring system. In occasional cases, tumor cells at an interface of tumor and stroma or infiltrating histiocytes/lymphocytes may appear positive but without significant staining within the more central part of the tumor. This edge effect is likely due to direct interaction between the tumor cell antigens and upregulation of PD-L1 expression by adjacent immune cells rather than the tumor cells. In cytologic specimen, when tumor cells are sparse, interpretation and scoring can be a challenge. Some cytologic preparations may contain an excess of histiocytes and tumor cells of comparable size, and it may be difficult to distinguish the two. Regardless of the nature of the biopsy, careful assessment of immunohistochemical stains, evaluation of positive and negative controls, as well as comparison with hematoxylin and eosin-stained tissue can minimize these difficulties [23].

### Resistance to immune checkpoint therapy

PD-1/PD-L1 blockade is a promising revolutionary cancer treatment strategy. Over the last decade, PD-1/PD-L1 inhibitors have been tested in a number of malignancies with considerable success. In addition, it has shown sustained survival benefits in multiple tumors and is at the forefront of cancer immunotherapy. Unfortunately, just as tumor cells can avoid immune evasion, several cancers may also evolve to resist PD-1/PDL1 blockade therapy. Thus, despite the potentially cure-like survival benefit, only a minority of patients experience a prolonged and sustained curative response to PD-1/PD-L1 blockade treatment [24].

Furthermore, the lack of long-term response to therapy is usually due to an acquired resistance that might eventually lead to cancer progression in patients with initially positive clinical response. Accordingly, the resistance to PD-1/PD-L1 blockade remains a significant challenge hindering its further application. Nevertheless, substantial efforts are underway to overcome the therapy resistance and to improve clinical response with minimal immune-mediated toxicity [24].

### The effect of chemotherapy on programmed cell death-ligand 1 (PD-L1) expression

A few studies have investigated the variation of PD-L1 immunostaining after neoadjuvant chemotherapy and explored the association between chemotherapy response, prognosis, and the increase or decrease of PD-L1 expression in cancer patients [25, 26]. Elevation of PD-L1 expression after neoadjuvant chemotherapy may be associated with altered chemotherapy response and progression-free survival. However, additional studies are required to evaluate the significance of these findings.

### Automation of PDL-1 interpretation

Manual scoring of PDL-1 IHC slides by pathologists may be a potential source of error. The increasing adoption of digital pathology and artificial intelligence in daily workflow of the laboratory provides an opportunity to leverage these tools towards improving the clinical value of PD-L1 IHC assays. Several recent studies have demonstrated that automated digital image analysis provides accuracy and consistency comparable to manual scoring. As such, image analysis scoring could serve as a crucial aid for pathologists in PD-L1 diagnostic testing [27–29].

In summary, considerable progress has been made towards understanding the role of immune checkpoint inhibitors in treating various cancers. PD-L1 testing for the selection of patients before administering therapy is a valuable technique, and its use is constantly expanding and evolving.

## Data Availability

Not applicable.

## Abbreviations

ATC: Activated cytotoxic T cells
MHC: Major histocompatibility complex
CTLA4: Cytotoxic T-lymphocyte-associated protein 4
TREG: Regulatory T cells
PD-1: Programmed cell death 1
PDL-1: Programmed cell death ligand 1
DC: Dendritic cells
N.K.: Natural killer
NSCLC: Non-small cell lung cancer
TPS: Tumor proportion score
CPS: Combined positive score
