# Correction: PD − L1 immunostaining: what pathologists need to know

**DOI:** 10.1186/s13000-022-01229-0

**Published:** 2022-06-11

**Authors:** Mohammed Akhtar, Sameera Rashid, Issam A. Al-Bozom

**Affiliations:** Department of Laboratory Medicine and Pathology, Hamad Medical Corporation, P.O. Box 3050, Doha, Qatar

**Correction: Diagn Pathol 16, 94 (2021)**

**https://doi.org/10.1186/s13000-021-01151-x**

Following publication of the original article [1], the authors noticed that the Fig. 10 is repeated as Fig. 11, with Fig. 11 being assigned figure number 12, and so on with Fig. 15 being omitted from the paper. Presented here are corrected Figs. 11, 12, 13, 14 and 15. The original article has been updated.

**Fig. 11:**
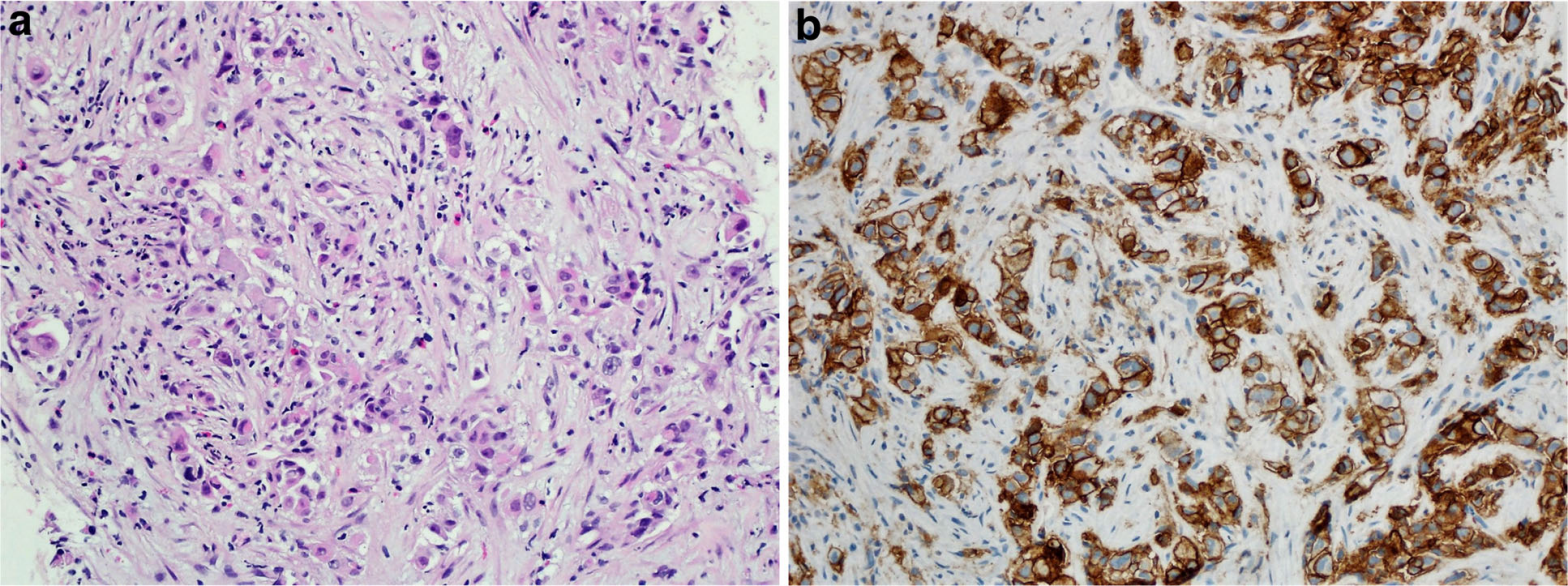
**A** Case of metastatic pulmonary adenocarcinoma to the liver. **B** Immunohistochemical staining for tumor cells, moderate to intense staining (2 + − 3+) for PD-L1 TPS: 90 (DAKO 22C3 antibody)

**Fig. 12:**
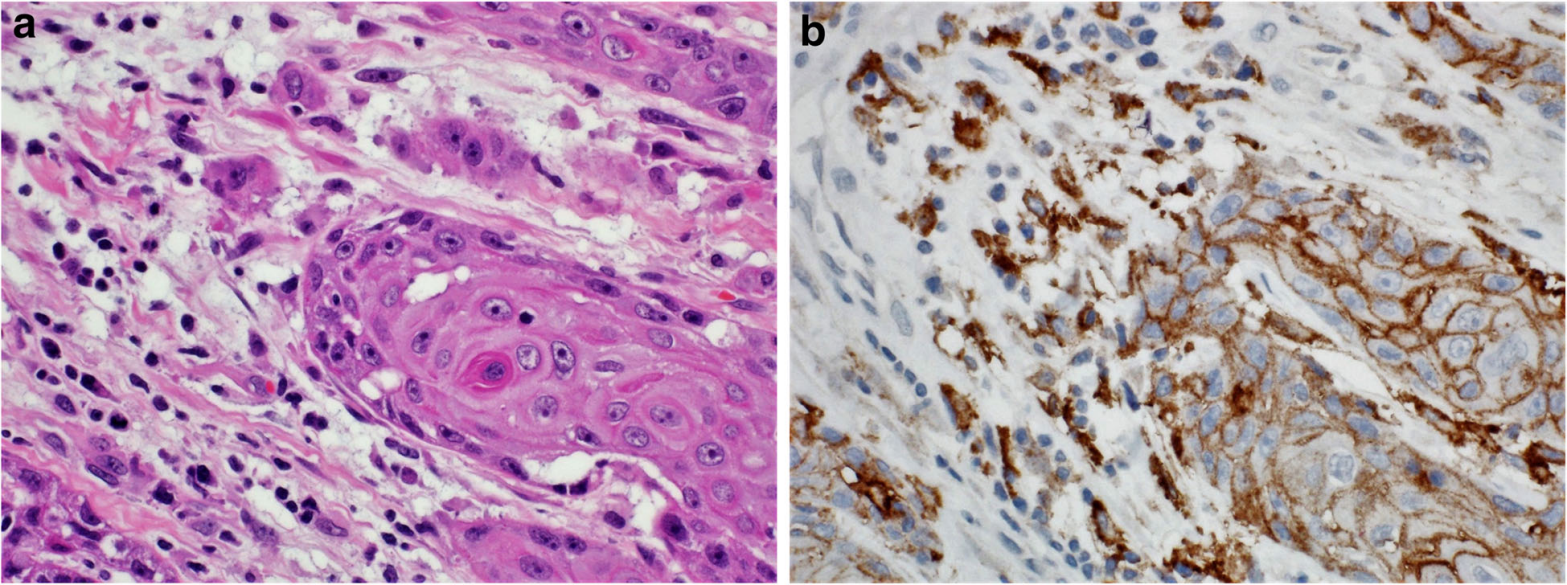
**A** Case of buccal mucosa squamous cell carcinoma. **B** Immunohistochemical staining for PD-L1 revealing staining of tumor cells (right side of the figure) and tumor immune cells (left side of the figure). CPS: 90 (DAKO 22C3 antibody)

**Fig. 13:**
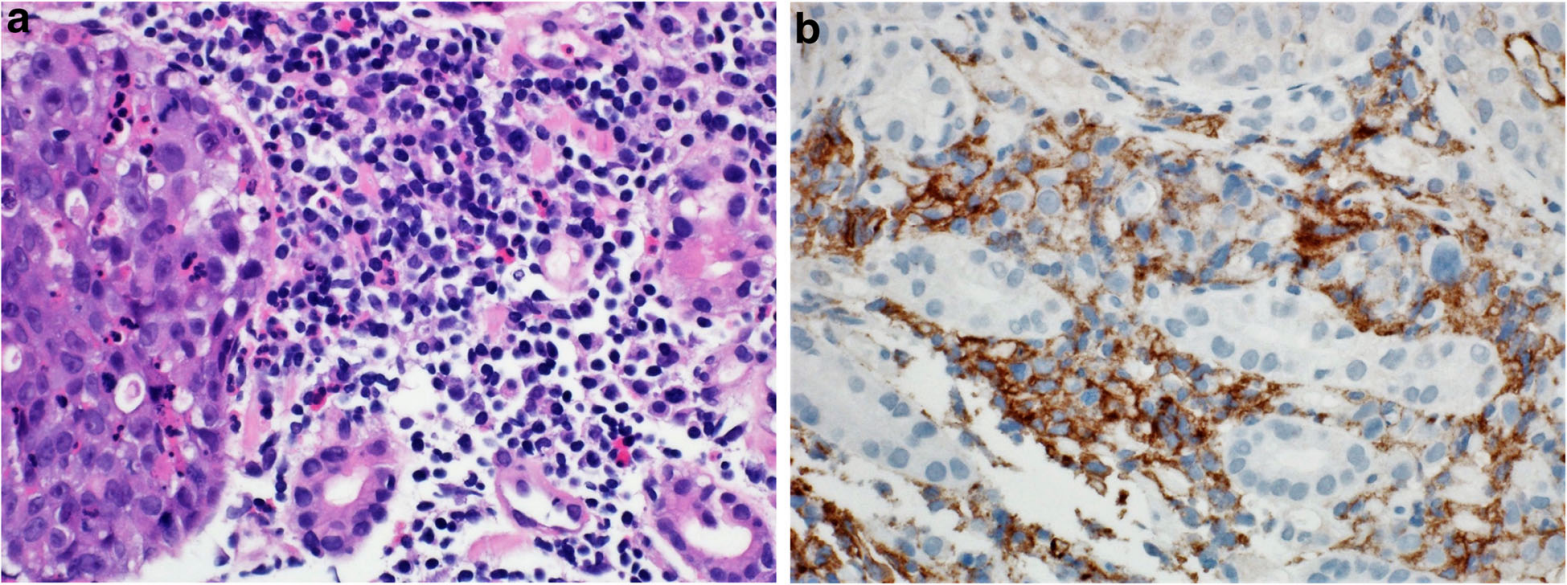
**A** Case of poorly differentiated gastric adenocarcinoma. **B** Immunohistochemical staining of the tumor showing staining of tumor immune cells while tumor cells are predominantly negative. CPS: 20. (DAKO 22C3 antibody)

**Fig. 14:**
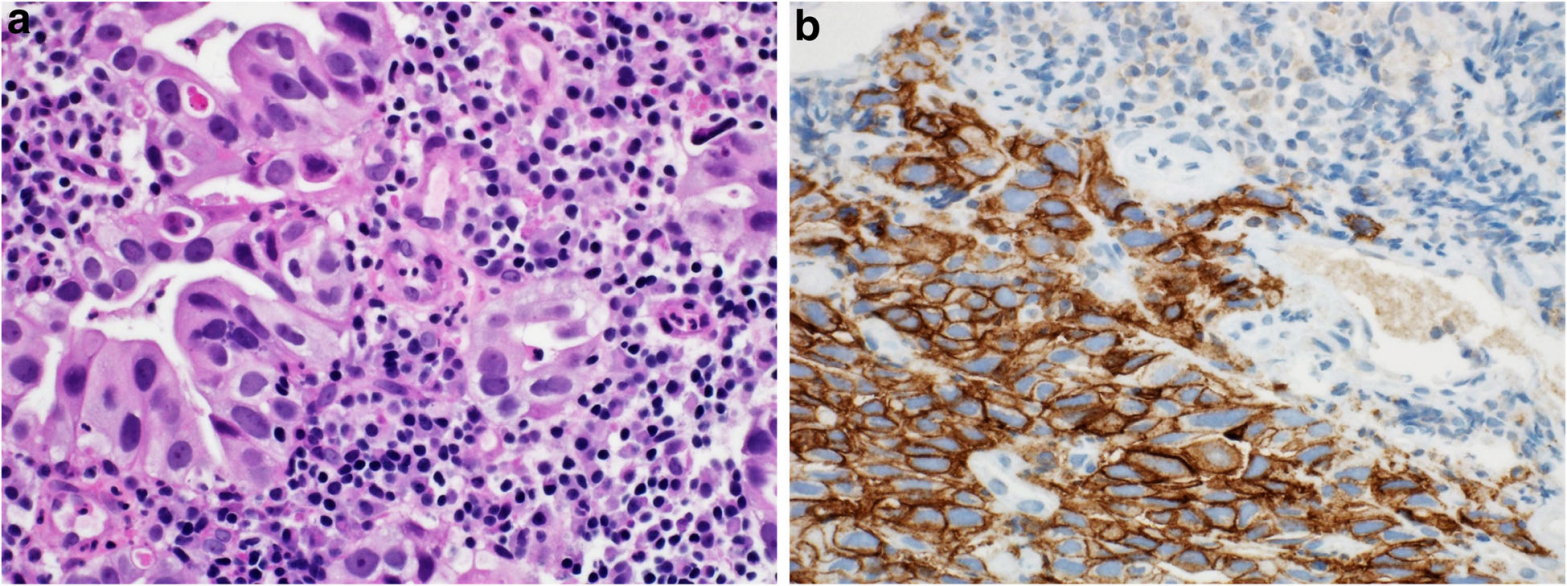
**A** Case of moderately differentiated gastric adenocarcinoma. **B** Immunohistochemical staining of the tumor in which only tumor cells are positive for PD-L1; while tumor immune cells are negative. CPS: 80. (DAKO 22C3 antibody)

**Fig. 15:**
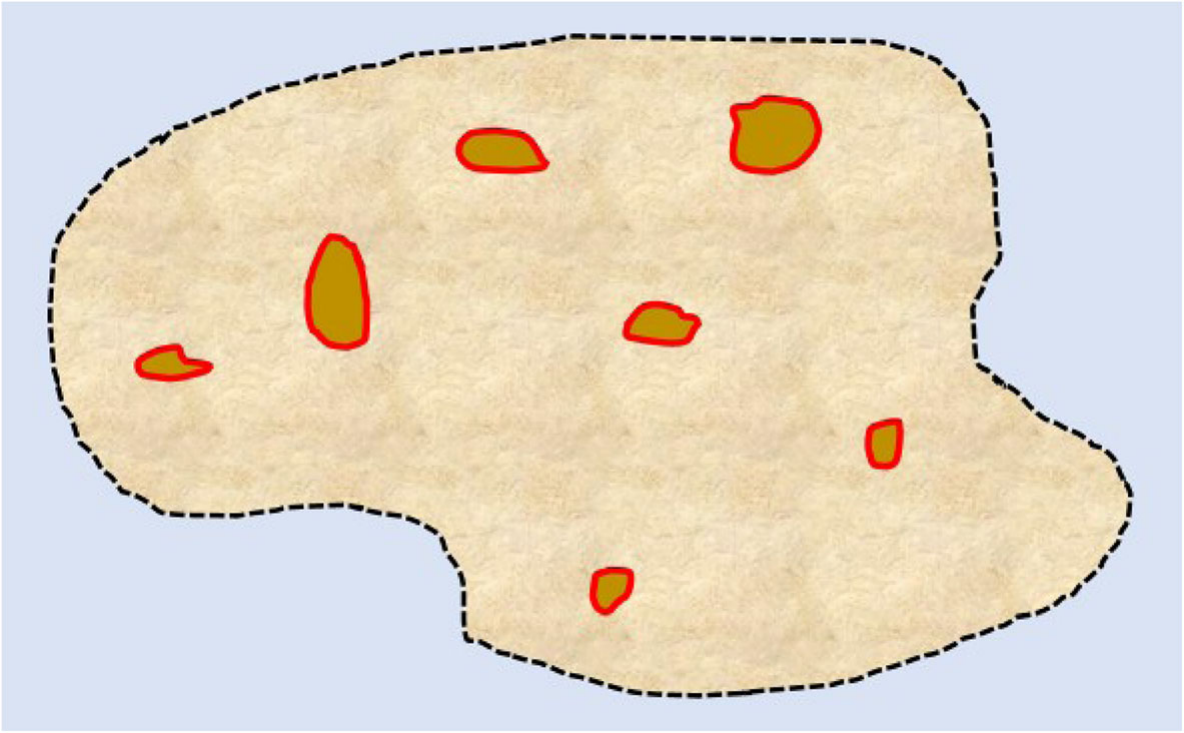
A diagram depicting expression of PD-L1 positive tumor infiltrating immune cells (marked by red border) in an area of tumor cells (marked by black dotted line). The proportion of tumor area occupied by PD-L1-positive immune cells of any intensity determines the immune cell (IC) score
